# SAVVY® (C31G) Gel for Prevention of HIV infection in Women: A Phase 3, Double-Blind, Randomized, Placebo-Controlled Trial in Ghana

**DOI:** 10.1371/journal.pone.0001312

**Published:** 2007-12-19

**Authors:** Leigh Peterson, Kavita Nanda, Baafuor Kofi Opoku, William Kwabena Ampofo, Margaret Owusu-Amoako, Andrew Yiadom Boakye, Wes Rountree, Amanda Troxler, Rosalie Dominik, Ronald Roddy, Laneta Dorflinger

**Affiliations:** 1 Family Health International, Durham, North Carolina, United States; 2 Komfo Anokye Teaching Hospital, Kwame Nkrumah University of Science & Technology, Kumasi, Ghana; 3 Noguchi Memorial Institute for Medical Research, University of Ghana, Accra, Ghana; 4 Duke Clinical Research Institute, Durham, North Carolina, United States; University of Stellenbosch, South Africa

## Abstract

**Objective:**

The objective of this trial was to determine the effectiveness of 1.0% C31G (SAVVY) in preventing male-to-female vaginal transmission of HIV infection among women at high risk.

**Methodology/Principal Findings:**

This was a Phase 3, double-blind, randomized, placebo-controlled trial. Participants made up to 12 monthly visits for HIV testing, adverse event reporting, and study product supply. The study was conducted between March 2004 and February 2006 in Accra and Kumasi, Ghana. We enrolled 2142 HIV-negative women at high risk of HIV infection, and randomized them to SAVVY or placebo gel. Main outcome measures were the incidence of HIV-1 and HIV-2 infection as determined by detection of HIV antibodies from oral mucosal transudate specimens and adverse events. We accrued 790 person-years of follow-up in the SAVVY group and 772 person-years in the placebo group. No clinically significant differences in the overall frequency of adverse events, abnormal pelvic examination findings, or abnormal laboratory results were seen between treatment groups. However, more participants in the SAVVY group reported reproductive tract adverse events than in the placebo group (13.0% versus 9.4%). Seventeen HIV seroconversions occurred; eight in participants randomized to SAVVY and nine in participants receiving placebo. The Kaplan-Meier estimates of the cumulative probability of HIV infection through 12 months were 0.010 in the SAVVY group and 0.011 in the placebo group (p = 0.731), with a hazard ratio (SAVVY versus placebo) of 0.88 (95% confidence interval 0.33, 2.27). Because of a lower-than-expected HIV incidence, we were unable to achieve the required number of HIV infections (66) to obtain the desired study power.

**Conclusions/Significance:**

SAVVY was not associated with increased adverse events overall, but was associated with higher reporting of reproductive adverse events. Our data are insufficient to conclude whether SAVVY is effective at preventing HIV infection relative to placebo.

**Trial Registration:**

ClinicalTrials.gov NCT00129532

## Introduction

Topical microbicides are designed to inhibit the sexual transmission of Human Immunodeficiency Virus (HIV) and other disease pathogens. They offer a female-controlled prophylactic option in situations where male condom use cannot be negotiated. Marketed chemical spermicides, which show some activity against sexually transmitted pathogens *in vitro*, have been evaluated as topical microbicides. However, no clinical studies have yet demonstrated that these products can prevent HIV infection, and spermicides with the surfactant nonoxynol-9 (N-9) have caused mucosal erosion and ulceration, which may increase the risk of HIV transmission [2]–[4].

Several *in vitro* studies [5] have suggested that C31G (SAVVY®, Cellegy Pharmaceuticals [formerly BIOSYN, Inc.], Huntington Valley, Pennsylvania) has a potent effect on enveloped HIV by disrupting the outer membrane. However, because its mechanism of action is similar to N-9 [6]; [7], some have raised concerns about the safety of SAVVY. In pre-clinical studies, SAVVY demonstrated minimal toxicity at 0.001% concentration, as measured by the cell proliferation assay, and no toxicity to mammalian cells at 0.0003%, as measured by the MTT assay [5]. In addition, 5 Phase 1 clinical studies, including a total of 121 women and 24 men exposed to SAVVY, have been conducted using three concentrations of SAVVY (0.5%, 1.0%, 1.7%). No serious or severe adverse events related to use of 1.0% SAVVY gel were reported during these studies [8]–[10]. We investigated the safety and effectiveness of 1.0% SAVVY in preventing HIV infection in a population of young, sexually active women at high risk for acquiring HIV infection.

## Methods

The protocol for this trial and supporting CONSORT checklist are available as supporting information; see Checklist S1 and Protocol S1.

### Participants

We conducted this randomized, double-blind, placebo-controlled trial between January 2004 and March 2006 in Accra and Kumasi, Ghana. We enrolled HIV-antibody negative, non-pregnant women 18 to 35 years old who were at risk of HIV infection because of having a mean of three or more coital acts per week and two or more sexual partners in the 3 months before screening. Study participants were recruited from areas within each city that were considered high HIV transmission areas, including markets, bars, and hotels. Although we did not specifically ask as part of the clinical trial procedures if the participants were sex workers, most exchanged sex for money. Special ethical considerations were taken into account because of the potential vulnerability of this population. We included women who agreed to: use study gel as directed and follow study procedures; report self-medication with antibiotics during study participation; and avoid use spermicides or other vaginal contraceptives or lubricants during the study. We excluded women who: were intending pregnancy; had a history of latex allergy; were injection drug users; or had gynecological conditions that could affect the safety or effectiveness of the study gel.

### Ethics

We developed strategies to protect the confidentiality and autonomy of the participants, increase/ensure comprehension of the informed consent and research methods, and promote access to resources and services during and after the trial. The study protocol and informed consent forms were approved by 1) the Committee on Human Research, Publications and Ethics, School of Medical Sciences, University of Science & Technology, Kumasi, Ghana, 2) Noguchi Memorial Institute for Medical Research IRB, University of Ghana, Legon, Ghana, and 3) the Protection of Human Subjects Committee, Family Health International, USA. All participants provided written informed consent in their preferred language. Illiterate participants were read the informed consent forms verbatim in the presence of a witness, and provided a mark or thumbprint in lieu of signature.

### Interventions

During recruitment, study staff explained the general purpose of the study and the eligibility requirements. If eligible, women were referred to one of two study clinics in Kumasi or Accra. At the screening visit, women were interviewed to confirm understanding and willingness to comply with study requirements, completed written informed consent, received HIV pretest and condom counseling, and underwent oral mucosal transudate (OMT) rapid HIV testing. All participants received HIV post-test counseling, physical and pelvic examinations (including vaginal wet mount to support the diagnosis for bacterial vaginosis, trichomoniasis, or vaginal candidiasis), urine pregnancy tests, and STI (syphilis, gonorrhea, and chlamydia) tests. Women with reactive OMT rapid HIV tests received ELISA to confirm HIV status. We asked potential participants to return 4 weeks after their screening visit to receive the results of their STI and confirmatory HIV tests, if applicable.

At this second visit, participants received a detailed explanation of study procedures, signed or marked a consent form for enrollment, received HIV counseling, provided urine for pregnancy testing, provided another OMT sample for HIV testing, and if eligible were randomized to receive either SAVVY or placebo. Study staff gave each eligible participant her first month's supply of study product after she had been randomized. Clinic staff counseled participants to use the gel vaginally before each act of sexual intercourse (and to insert a second dose if more than one hour had elapsed between the first application and sexual intercourse), emphasized that the gel had unknown effectiveness for preventing HIV, distributed condoms, and counseled participants to use condoms for all sexual contacts with all partners. The informed consent form stated that: “We do not know the effects and safety of the gel during pregnancy. Pregnant women may not join this study. If you become pregnant during the study you should tell your study doctor or nurse right away. Your study gel will be stopped and the study doctor will discuss your choices with you.”

At each monthly follow-up visit, participants underwent OMT HIV and pregnancy testing, AE assessment, STI risk reduction counseling, and study product and condom re-supply. Participants responded to structured questionnaires on their interval sexual behavior (including coital activity, and gel and condom use), experience using the gel, and were reminded of study concepts discussed during the informed consent process. If clinically indicated, participants underwent physical examination and STI testing. Study staff documented whether product use was interrupted temporarily or permanently for any of the following reasons: participant ran out of supplies, investigator withdrew study product in the interest of the safety and well being of the participant, positive pregnancy test result, or confirmed HIV infection. Pregnant women were allowed to resume study product use after pregnancies had ended. Study staff referred participants infected with HIV during the study to local HIV-related psychological, social, and medical services (such as viral load, CD4 level, and HIV resistance testing) as well as antiretroviral drug therapy when needed. If a participant missed a scheduled follow-up appointment, study staff made up to 3 attempts to contact that participant and reschedule the appointment (ideally to occur within 2 weeks of the original appointment). If the participant could not be located after 3 attempts, no further efforts were made to find her, but her file remained open until study closeout. If the participant did not return to the study before the study was closed, she was considered lost to follow-up. The “lost to follow-up” designation was not made for any participant until the closing date of the study.

### Objective

The objective of this trial was to determine the effectiveness of 1.0% C31G in preventing male-to-female vaginal transmission of HIV infection among women at high risk.

### Outcomes

The primary measure of effectiveness was infection with HIV-1 or HIV-2, measured by detecting antibodies in oral mucosal transudate (OMT) (OraQuick® ADVANCE Rapid HIV-1/2 Antibody Test, Orasure Technologies) and confirmed by an enzyme-linked immunosorbent assay (ELISA) (Genetic Systems™ HIV-1/HIV-2 Plus O ELISA from BioRad) and/or Western Blot (Genetic Systems™ HIV-1 Western Blot, BioRad) from a finger prick or serum specimen. We evaluated safety by comparing the incidence of adverse events (AEs) including pelvic exam findings and sexually transmitted infections (STIs).

### Sample Size

We estimated that a sample size of 2142 participants (1,071 in each treatment group) would give us 80% power to detect a 50% difference in the HIV infection rate (two-sided log-rank test, α = 0.05 significance level) between the two groups. We assumed the rate of HIV infection in the control group to be 5/100 person-years and loss to follow-up to be 20%; approximately 66 total HIV infections were needed to achieve the desired power. Our protocol included plans for assessing (in a blinded manner) whether additional participants would be needed to observe the required 66 events.

### Randomization and Blinding

We randomized enrolled participants into either the SAVVY or placebo arm using a 1∶1 allocation ratio. A statistician not otherwise involved with the study developed the allocation sequence using a computer random number generator and randomly varied permuted-blocks of 12, 18, and 24. Six label colors (three SAVVY and three placebo) were used to differentiate the otherwise identically packaged gels. Randomization was stratified by study site. We used sequentially numbered, sealed opaque envelopes to assign participants to one of six color groups after they had qualified for the study and signed the consent form. The randomization envelopes were maintained in a secure office and were not available to study staff until the moment of randomization. Each randomization envelope was used only once. Participants, field study staff, monitors, statisticians, and other FHI staff involved in the trial were not aware of which gel colors were associated with SAVVY or placebo. Both SAVVY and placebo gels were clear, with similar viscosity and pH, dispensed in 3.5 mL doses with identical applicators.

The placebo gel was formulated to minimize any possible effects—negative or positive—on study endpoints. It was isotonic to avoid epithelial cell swelling or dehydration, and formulated at a pH of 4.4 but with minimal buffering capacity. When mixed with an equal volume of semen, the placebo gel induced only a trivial decrease in semen pH (from 7.8 to 7.7). The placebo gel contained hydroxyethylcellulose as a gelling agent, and its viscosity was comparable to that of SAVVY. Hydroxyethylcellulose does not have anti-HIV properties. The gel also contained sorbic acid as a preservative; sorbic acid has no anti-HIV activity and is readily metabolized by human cells.

### Statistical Methods

For the primary effectiveness analysis we compared the probability of HIV infection for the SAVVY and placebo gel groups using a two-sided site-stratified, exact log-rank test (StatXact). We calculated Kaplan-Meier estimates of HIV infection probabilities by treatment group, pooled across sites. We used a proportional hazards regression model to estimate the hazard ratio, along with a 95% confidence interval, comparing the SAVVY group to the placebo group for HIV incidence, controlling for site. Each HIV infection onset date was estimated as the midpoint between the date of the first positive test result and the previous, negative test date. A right censoring time of 380 days was applied. Because the trial was terminated well before reaching the number of HIV infections targeted for pre-planned tests of effectiveness (i.e., before any of the type I error was to be spent), p-values for analyses of effectiveness should be interpreted as descriptive statistics. We calculated exact 95% confidence intervals for the relative risk of pre-specified priority AEs within system organ classes under a Poisson assumption for the event rates in each treatment group (StatXact). We compared proportions of women with any pelvic exam findings or STDs between treatment groups with a two-sided Mantel-Haenszel Chi-Square Test stratified by site at the 0.05 significance level.

All primary and most secondary analyses were either conducted on the Effectiveness Population which is the subset of the Intent-to-Treat (ITT) Population for whom at least one post-enrollment HIV evaluation is available, or the Safety Population which is the subset of the ITT Population who ever returned after enrollment. The Evaluable Population includes the same participants as the Effectiveness Population but excludes all data collected from a participant after her first documented interruption of product use.

Our data monitoring plan specified that the independent Data Monitoring Committee (DMC), with access to treatment assignments, would review AEs and primary safety and HIV seroconversion data twice, after approximately 16 and 33 events, respectively. However, testing for early evidence of effectiveness was scheduled only to occur at the second of these two planned looks and later when the target total number of events (66) was obtained (i.e., the first look at the HIV seroconversion data was to review the data for signs of harm) .

Monitors (trained in Good Clinical Practice) visited sites regularly to review study eligibility, informed consent, protocol compliance, laboratory procedures, source documents, product accountability, and AEs. We attempted to get original hospital records, when available, for serious adverse events (SAEs) and deaths.

## Results

### Recruitment, Participant Flow, Numbers Analyzed and Baseline Characteristics

**Figure 1:**
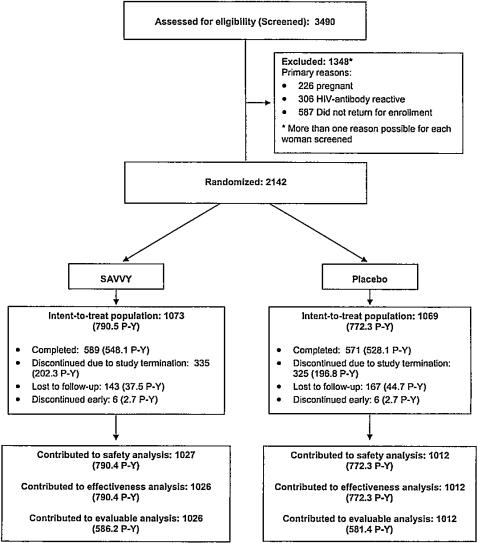
Participant Trial Flow Diagram (P-Y, person years).

Participants were recruited and enrolled into this study for 15 months, starting February 2004. A total of 3,490 women were screened, from which we enrolled 2,142 participants into the study (Figure 1). The primary reasons potential participants were not enrolled were because they failed to return for enrollment, were HIV-infected at baseline, or were pregnant. Overall loss to follow-up after enrollment was approximately 15% (n = 310). The loss-to-follow-up rate was highest during the first month of study participation, during which 40% of the overall loss to follow-up (123 of 310 participants) occurred. Among these were 103 participants who never returned after their enrollment visit and were therefore excluded from the primary safety analyses.

The first scheduled DMC safety review was repeated because errors were found in the initial HIV seroconversion data due to false positive ELISA testing. At the third DMC safety review meeting, when there were 16 seroconversions in the database, we informed the DMC that the study statistician estimated that approximately 3,500 additional participants (beyond the 2,142 planned sample size) would be needed to achieve the required number of HIV infections (66) to obtain the desired study power. In light of this formidable challenge, we requested that the DMC review the available outcome data by group (along with results of conditional power calculations under a range of assumptions about the true effect size) and provide a recommendation as to whether the trial should continue or be terminated early. The DMC recommended that the trial be stopped; we thus decided to prematurely terminate the study in November 2005.

Due to premature termination of the study, 660 participants exited the study before completing their Month 12 visit (Figure 1). Twelve additional participants (0.6%) discontinued early (6 in each treatment group), including one participant in each of the treatment groups who died during follow-up. Other than the deaths, which were unrelated to product use, no participants withdrew early due to a medical reason. Most enrolled participants in both groups were young (mean age 22.7), unmarried, and had fewer than 9 years of education. Other demographic characteristics and medical history were also similar (Table 1).

**Table 1:** Baseline Characteristics (ITT Population)

	SAVVY (N = 1073)	Placebo (N = 1069)
**Characteristic**		
Age	22.7 (3.6)	22.7 (3.6)
Marital status		
Unmarried, not cohabiting	946 (88.2)	945 (88.4)
Education		
≤ 9 years	831 (77.4)	812 (76.0)
Pregnancy history		
Ever pregnant	782 (72.9)	782 (73.2)
Number of pregnancies	2.1 (1.4)	2.1 (1.4)
Number of vaginal deliveries	0.9 (1.0)	0.9 (0.9)
Contraceptive use		
Condom	496(46.2)	516 (48.3)
None	423 (39.4)	397 (37.1)
Oral	122 (11.4)	125 (11.7)
Injectable	23 (2.1)	22 (2.1)
IUD	5 (0.5)	5 (0.5)
Other	4 (0.4)	4 (0.4)
History of STI	182 (17.0)	199 (18.6)
Previous spermicide use	91 (8.5)	77 (7.2)
Douching	578 (53.9)	555 (51.9)

Data reported as N(%) or mean (SD); SD = standard deviation, STI = sexually transmitted infection

### Sexual Behavior

At enrollment participants reported a mean coital frequency of 9–10 acts per week (SAVVY 9.5; placebo 8.9) and a mean of 5.8 different partners in the last 30 days (in both groups). Approximately 2% of the participants (SAVVY 1.8%; placebo 2.5%) reported having anal sex in the previous 30 days. During follow-up, participants consistently reported a mean of 7 coital acts per week, with a mean of 5–6 sexual partners in the previous 30 days. Self-reported condom use increased from 40% during the last coital act before screening to over 89% at the first follow-up visit, remaining relatively constant throughout follow-up.

Although pregnant participants are included in the intent-to-treat analysis, we asked these participants to stop gel use upon a positive pregnancy test (they could return to product use after a negative pregnancy test). A total of 383 participants in the SAVVY group and 386 participants in the placebo group became pregnant at least once during the study. The Kaplan-Meier pregnancy probability at 12 months was 42.5 per 100 person-years in the SAVVY group and 43.7 per 100 person-years in the placebo group. Of the women who became pregnant during the study, most (79%) were pregnant only once. However, approximately 19% were pregnant twice, and almost 2% were pregnant three times during the course of the study. The median amount of time without gel use due to pregnancy (among the participants who became pregnant) was approximately 2 months, resulting in 151 person-years (or 10% of the total person-time) off product due to pregnancy. Of the 942 total pregnancies detected during the study (among 769 participants), pregnancy outcome information was available for 606 pregnancies. Other than 13 spontaneous abortions (4 in participants receiving SAVVY and 9 in participants receiving placebo), no abnormal outcomes were reported. A total of 40 live births (all normal) were reported to have occurred among study participants.

### Product Use

Participants reported that they used the gel for an average of 75% and 77% of coital acts in the SAVVY and placebo groups, respectively, and reported condoms use for 89% and 90% of coital acts in the SAVVY and placebo groups, respectively. They reported using both gel and condoms for 70.1% and 71.7% of acts in the SAVVY and placebo groups, respectively. Participants reported using neither gel nor condoms for 6.2% and 5.3% of acts in the SAVVY and placebo groups, respectively. Gel use decreased with time in both groups (from 86–87% during Month 1 to 67–71% during Month 12), whereas condom use was more consistent. From these data, we calculated that SAVVY or placebo gel was used alone (*i.e.,* without a condom) for approximately 5% of all vaginal acts and that condoms were used alone (*i.e.,* without gel) for approximately 16% of all vaginal acts. We also calculated that SAVVY or placebo gel was used for 81% of vaginal acts during which a condom was also used, but for only 43–47% of acts where no condom was used (Table 2).

**Table 2:** Overall Estimates of Gel and Condom Use at Follow-Up by Treatment Groups*

	Overall	
	SAVVY N = 1021	Placebo N = 1008
**Mean percentage of vaginal sex acts in the last 7 days with study gel**		
Mean (SD)	75.0 (24.0)	77.0 (23.1)
Range (median)	0–100 (81.6)	0–100 (83.0)
**Mean percentage of vaginal sex acts in the last 7 days with a condom**		
Mean (SD)	88.6 (16.5)	89.5 (15.9)
Range (Median)	0–100 (94.9)	0–100 (96.0)
**Mean percentage of vaginal sex acts in the last 7 days with both the study gel and a condom**		
Mean (SD)	70.1 (26.5)	71.7 (25.8)
Range (Median)	0–100 (76.5)	0–100 (77.8)
**Mean percentage of vaginal sex acts in the last 7 days with neither a condom nor study gel**		
Mean (SD)	6.2 (11.8)	5.3 (10.7)
Range (Median)	0–100 ( 0.0)	0–100 ( 0.0)
**Mean percentage of vaginal sex acts in the last 7 days with study gel only (without a condom)**		
Mean (SD)	5.1 (17.6)	5.2 (17.9)
Range (median)	0–100 ( 0.0)	0–100 ( 0.0)
**Mean percentage of vaginal sex acts in the last 7 days with a condom only (without study gel)**		
Mean (SD)	16.4 (32.4)	16.5 (32.7)
Range (Median)	0–100 ( 0.0)	0–100 ( 0.0)
**Mean percentage of vaginal sex acts in the last 7 days with study gel *and a condom***		
Mean (SD)	80.8 (35.8)	80.8 (36.2)
Range (Median)	0–100 ( 100)	0–100 ( 100)
**Mean percentage of vaginal sex acts in the last 7 days with study gel *when a condom is not used***		
Mean (SD)	43.1 (47.3)	46.9 (47.9)
Range (Median)	0–100 ( 0.0)	0–100 (29.3)

* For each participant and variable of interest (e.g., percentage of vaginal acts where study gel was used in the last 7 days prior to the follow-up visit), we first calculated the participant's mean value of the variable of interest across all of their follow-up visits. (Follow-up visits where women reported 0 or a missing number of vaginal sex acts in the last 7 days are excluded from the calculation of a participant's mean value.) The mean, SD, range, and median values of the distributions of these mean values were then obtained for each treatment group.

### Safety: Adverse Events

Approximately one-third of participants in both groups had adverse events (33% in the SAVVY group and 29% in the placebo group), with no significant differences in the overall frequency of AEs between treatment groups. The most frequently reported AEs included: malaria, abdominal pain, headache, genital pruritus, vaginal candidiasis, general pain, bacterial vaginitis, and vaginal discharge. No significant differences in frequency of AEs occurred between treatment groups. However, more participants in the SAVVY group had reproductive tract AEs, with an incidence of 14.5 per 100 person-years in the SAVVY group versus 10.9 per 100 person-years in the in the placebo group (rate ratio = 1.33, 95% confidence interval 0.99, 1.80, [Table 3]). The most frequently occurring AEs with the reproductive tract were genital pruritus, vaginal candidiasis, vaginal discharge, bacterial vaginitis, and vulvovaginitis.

**Table 3:** Selected Priority Adverse Events

System Organ Class/ Preferred Term	SAVVY (N = 1027 )				Placebo (N = 1012 )				Rate Ratio (95% CI), SAVVY vs. Placebo
	No. of Events	No. of Women	% of Women	IR* (per 100 person years)	No. of Events	No. of Women	% of Women	IR* (per 100 person years)	
**Reproductive system and breast disorders**	**140**	**107**	**10.4**	**14.5**	**120**	**80**	**7.9**	**10.9**	**1.33 (0.99, 1.80)**
Amenorrhoea	3	3	0.3	0.4	0	0	0.0	0.0	INF
Cervicitis	0	0	0.0	0.0	1	1	0.1	0.1	0.00
Dysmenorrhoea	6	6	0.6	0.8	4	4	0.4	0.5	1.47
Dyspareunia	1	1	0.1	0.1	1	1	0.1	0.1	0.98
Genital abscess	1	1	0.1	0.1	2	2	0.2	0.3	0.49
Genital pruritus female	28	27	2.6	3.5	23	20	2.0	2.6	1.32
Genitourinary tract gonococcal infection	0	0	0.0	0.0	1	1	0.1	0.1	0.00
Menorrhagia	1	1	0.1	0.1	2	2	0.2	0.3	0.49
Menstruation irregular	7	7	0.7	0.9	7	6	0.6	0.8	1.14
Ovulation pain	1	1	0.1	0.1	1	1	0.1	0.1	0.98
Pelvic inflammatory disease	5	5	0.5	0.6	4	4	0.4	0.5	1.23
Post coital bleeding	1	1	0.1	0.1	1	1	0.1	0.1	0.98
Vaginal abscess	3	3	0.3	0.4	1	1	0.1	0.1	2.94
Vaginal burning sensation	0	0	0.0	0.0	3	2	0.2	0.3	0.00
Vaginal candidiasis	29	28	2.7	3.6	30	28	2.8	3.7	0.98
Vaginal discharge	16	16	1.6	2.0	13	11	1.1	1.4	1.43
Vaginal erythema	4	3	0.3	0.4	0	0	0.0	0.0	INF
Vaginal haemorrhage	0	0	0.0	0.0	1	1	0.1	0.1	0.00
Vaginal laceration	0	0	0.0	0.0	2	2	0.2	0.3	0.00
Vaginal pain	2	2	0.2	0.3	1	1	0.1	0.1	1.96
Vaginitis bacterial	19	19	1.9	2.4	16	14	1.4	1.8	1.33
Vulvovaginal ulceration	1	1	0.1	0.1	1	1	0.1	0.1	0.98
Vulvovaginitis	11	11	1.1	1.4	5	5	0.5	0.6	2.16
Vulvovaginitis trichomonal	1	1	0.1	0.1	0	0	0.0	0.0	INF
**Gastrointestinal disorders**	**85**	**73**	**7.1**	**9.7**	**94**	**76**	**7.5**	**10.4**	**0.93 (0.67, 1.30)**
**Pregnancy, puerperium and perinatal conditions**	**7**	**7**	**0.7**	**0.9**	**13**	**12**	**1.2**	**1.6**	**0.57 (0.19, 1.57)**
**Renal and urinary disorders**	**15**	**12**	**1.2**	**1.5**	**18**	**17**	**1.7**	**2.2**	**0.69 (0.30, 1.53)**

* Incidence Rate

In post hoc analyses that examined subgroups defined by self-reported frequency of gel use, we found a greater treatment group difference in the incidence of reported reproductive system and breast disorders (17.3 per 100 person-years in the SAVVY group and 10.1 per 100 person-years in the placebo group) among the subgroup of participants whose self-reported gel use was at or below the overall median than among the subgroup of participants whose self-reported gel use was above the overall medium. Among participants whose self-reported gel use was at or below the overall median the rate ratio was 1.71 (95% confidence interval = 1.13–2.61). Similarly, the rate ratio was 1.64 (95% confidence interval = 1.09–2.50) for the subgroup of participants with self-reported sexual frequency at or below the overall median (17.4 per 100 person-years in the SAVVY group and 10.6 per 100 person-years in the placebo group).

Participants underwent pelvic examination and STI testing during follow-up only if clinically indicated. During follow-up, 121 participants had pelvic examinations (63 in the SAVVY group and 58 in the placebo group). No significant differences were seen between treatment groups for bacterial vaginosis, trichomoniasis, or vaginal candidiasis on saline wet mounts.

Twenty-two SAEs were reported; 15 by participants in the SAVVY group and 7 by participants in the placebo group. Only one, gonorrhea, was classified by the investigator as possibly related to the study product (the participant had received placebo). Two deaths occurred during the course of the study. One was suspected to be due to possible sickle cell crisis (the participant had been randomized to SAVVY), and one was due to viral hepatitis complicated by hepatic encephalopathy (the participant had been randomized to placebo). No participant was asked to discontinue study drug due to an AE.

### Effectiveness: HIV Incidence

The overall HIV incidence rate was 1.09 per 100 person years (95% confidence interval 0.63, 1.74). In the Effectiveness Population, we observed 17 HIV seroconversions, 8 in the SAVVY group and 9 in the placebo group. The Kaplan-Meier estimates of the cumulative probability of HIV infection at 12 months were 0.010 in the SAVVY group and 0.011 in the placebo group (log-rank p = 0.731), with a hazard ratio (SAVVY versus placebo) of 0.88 (95% confidence interval 0.33, 2.27). Of these 17 seroconversions, 9 were in Accra and 8 occurred in Kumasi. Fifteen seroconversions (7 SAVVY; 8 placebo) occurred in participants younger than 25 years. Ten (5 SAVVY; 5 placebo) of the seroconverters had baseline coital frequency at or below the median and 7 (3 SAVVY; 4 placebo) had baseline coital frequency above the median.

When we repeated the analysis in the Evaluable Population, we observed 11 HIV infections (4 in the SAVVY group; 7 in the placebo group). The Kaplan-Meier estimates of the cumulative probability of HIV infection at 12 months were 0.007 in the SAVVY group and 0.012 in the placebo group, with a hazard ratio of 0.57 (95% confidence interval of 0.17 to 1.94).

## Discussion

### Interpretation and Overall Evidence

We stopped this study prematurely following recommendations of an independent DMC because the HIV incidence among enrolled participants was substantially lower than expected; we therefore could not evaluate the effectiveness of SAVVY in preventing HIV as intended. Due to the small number of events available for analysis, this trial was unable to meet the objective of accessing the effectiveness of SAVVY in preventing male-to-female transmission of HIV. To date, no randomized clinical studies have identified a microbicide that is effective at preventing HIV infection [11].

Lower-than-expected HIV incidence has been an increasing problem in HIV prevention trials and the lower-than-expected HIV incidence seen in this trial may have been due in part at least 3 factors: 1) a greater-than-expected effect of trial risk reduction measures such as condom use, 2) participants who join clinical trials may be more inclined to safer behavior than those in their community who do not participate, and 3) an inaccurate estimate of trial incidence due to changes in the local epidemic. The incidence rate for this trial was estimated from our experience in earlier trials in a similar population and was not specifically measured in each population before starting the study.

Ensuring high product adherence is another important challenge in the successful conduct of HIV prevention studies. Low product use due to such factors as pregnancy, missed visits, or participant's choice compromises study power. Although participants reported using the gel during approximately 76% of coital acts, our calculations show that participants were more likely to use gel if a condom was also used than when a condom was not used (>80% gel use with condoms versus <50% gel use without condoms). Since the percent of gel use among risky acts (*i.e.*, with an HIV-infected partner) without condom use is a major determinant of the overall level of effectiveness that can be observed for a truly efficacious product, unless gel use can be increased, studies designed to detect a 50% effect may not be able to identify truly efficacious products[12]. We observed a pregnancy probability at 12 months of 42.5 per 100 person-years in the SAVVY group and 43.7 per 100 person-years in the placebo group. If further studies demonstrate that SAVVY is an effective contraceptive method in other populations, the lack of a difference between the observed pregnancy rates for the SAVVY and placebo groups in our study may indicate that the level of gel adherence was even lower than the participants reported.

The likelihood of pregnancy in reproductive-age women having multiple sexual encounters is quite high even with relatively high condom use. Using Wilcox's estimates of the probability of pregnancy resulting after one or more unprotected act for each day of the menstrual cycle (assuming 30 days per cycle and 12 cycles per year), we expect that if women have two days with unprotected acts on average during each cycle (*e.g*., 90% condom use, 20 acts per cycle, and no other contraceptive method use), the 12-month cumulative pregnancy probability would be 51%. Therefore, the pregnancy probability of 43% at 12 months observed during this study is not inconsistent with what the participant's reported–89% condom use and a coital frequency of 30 acts per month. Unless adequate and well-controlled safety studies of microbicides in pregnant women are conducted prior to the start of future HIV prevention studies thereby allowing women who become pregnant to remain on study product, investigators should consider mandating that all female participants who have reproductive potential must be using an effective non-barrier contraceptive such as depot medroxyprogesterone acetate (DMPA), combined oral contraceptive pills (COCs), an intrauterine device (IUD), or a contraceptive implant at least at the time of enrollment.

We did not observe a significant difference in the overall rate of adverse events between participants receiving SAVVY and those receiving placebo. The frequency of adverse events in the reproductive tract/breast disorders (specifically symptoms of vaginal or vulvar irritation) appeared higher in the SAVVY group, but further subgroup analyses revealed no evidence that the effect of SAVVY use was more pronounced among women with more frequent exposure to the product. Whether the observed increased risk for these events among the SAVVY users in the subset of women who had fewer than average sexual acts and/or fewer gel uses is a chance finding, a result of confounding due to subgrouping by a post-randomization factor, or evidence of an actual negative association between treatment effect and frequency of intercourse, is unclear.

One limit of our study is generalizability. Although we did not specifically recruit sex workers, women in our study averaged a coital frequency of 7 acts per week and 5–6 different partners in the last month. Thus conclusions should be interpreted cautiously with regard to other populations.

The availability of study product did not appear to result in increased risk taking behaviors among participants. During enrollment, participants reported a mean of 9 coital acts per week with a mean of 6 sexual partners in the previous 30 days. During follow-up, participants reported a mean of 7 coital acts per week, with a mean of 5–6 sexual partners in the previous 30 days. Reported condom use during follow-up increased from 40% during the last coital act prior to screening to over 89% at the first follow-up visit and remained relatively constant throughout the entire follow period.

### Conclusion

As a new HIV prevention approach, microbicides could be used with other prevention strategies such as condoms to reduce the number of people who become infected with HIV. In our study, SAVVY was not associated with increased adverse events overall relative to placebo, but was associated with higher reporting of reproductive adverse events. Our data are insufficient to conclude whether SAVVY is effective at preventing HIV infection relative to placebo. Phase III trials with larger numbers of seroconversions are needed to determine the effectiveness, safety, and feasibility of using microbicides for prevention of HIV infection in women.

## Supporting Information

Checklist S1 CONSORT Checklist
(0.05 MB DOC)

Protocol S1 Trial Protocol
(0.74 MB DOC)
